# Beyond vision loss: the impact of glaucoma on the brain

**DOI:** 10.3389/fcell.2025.1588561

**Published:** 2025-07-31

**Authors:** Trinita Jude Hamilton, Bettina E. Kalisch

**Affiliations:** Department of Biomedical Sciences, University of Guelph, Guelph, ON, Canada

**Keywords:** glaucoma, neural degeneration, cognitive impairment, optic disc, retinal ganglion cell

## Abstract

Glaucoma, a leading cause of irreversible blindness, is characterized by optic disc cupping and retinal ganglion cell (RGC) degeneration. Recent research highlights the broader impacts of glaucoma on the brain. Transsynaptic neural degeneration extends the RGC damage through the visual pathway and various other regions in the brain, leading to structural and functional changes. These neurodegenerative effects may result in cognitive impairment, affecting patients’ daily activities and quality of life. Understanding the connection between glaucoma, the brain, and cognition is essential for intervention and developing comprehensive treatment strategies that address visual and neurological impairments, ultimately improving patient outcomes. This review examines the existing literature on the links between glaucoma pathology and the brain and explores the impacts on cognition and quality of life.

## 1 Introduction

Glaucoma is an optic neuropathy and a leading cause of global irreversible blindness (Tham et al., 2014; Kang and Tanna, 2021). The disease is initially asymptomatic, progressing from peripheral to central vision loss as severity increases (Quigley, 2011; Kang and Tanna, 2021). Although high intraocular pressure (IOP) is a major risk factor for glaucoma, some individuals develop glaucoma with a normal IOP, suggesting an underlying etiology (Kim and Park, 2016). Growing research on the eye-brain connection in neurodegenerative disorders highlights the involvement of central nervous system (CNS) changes in glaucoma pathology (Arrigo et al., 2021). As glaucoma involves irreversible vision loss, a clearer understanding of the disease may help develop preventative and therapeutic strategies. This literature review examines how glaucoma impacts the CNS, cognitive function, and quality of life.

## 2 Glaucoma

### 2.1 Pathology

**FIGURE 1:**
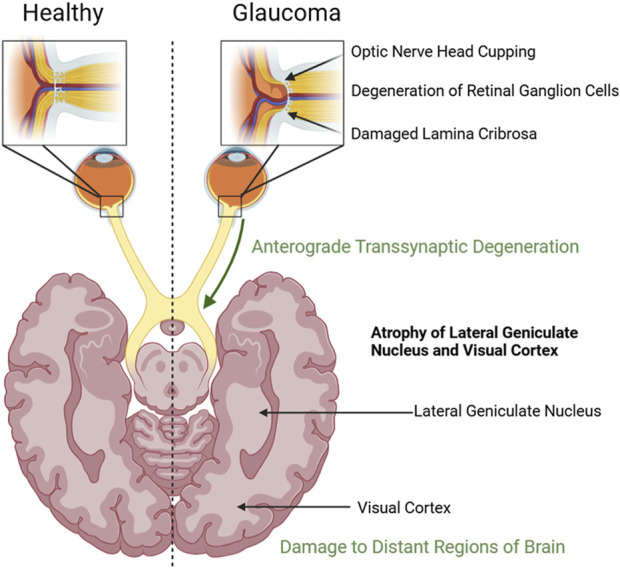
Comparison of a healthy eye and brain to one with glaucomatous damage. The glaucoma eye exhibits retinal ganglion cell degeneration, lamina cribrosa damage, and optic disc cupping. The glaucomatous brain has atrophy of the lateral geniculate nucleus, visual cortex, and distant regions. Created using Biorender.com.

Glaucoma is characterized by apoptotic degeneration of retinal ganglion cells (RGCs) and their axons, which form the optic nerve responsible for transmitting visual information from the retina to the brain (Figure 1) (Kang and Tanna, 2021). This leads to retinal nerve fiber layer (RNFL) thinning and optic disc cupping (Quigley, 2011; Alasil et al., 2014). High IOP is a significant risk factor for optic cup enlargement, as it damages the lamina cribrosa, resulting in disrupted structural integrity and reduced metabolic support for RGC axons, triggering apoptosis (Kang and Tanna, 2021). IOP is regulated by the balance of aqueous humor production and outflow within the anterior chamber of the eye, and lowering IOP through laser, pharmaceutical, or surgical treatments remains the only proven option to slow disease progression (Kang and Tanna, 2021; Kass et al., 2002).

### 2.2 Epidemiology

Glaucoma is classified as open-angle or angle-closed based on the cause of impaired aqueous humor drainage (Quigley, 2011). In open-angle glaucoma, the anterior chamber angle remains open, but drainage is impaired, whereas angle-closure glaucoma involves a clinically visible obstruction reducing or blocking outflow (Kang and Tanna, 2021). Glaucoma can be further categorized as primary, with no identifiable cause for high IOP, or secondary, resulting from conditions such as trauma, inflammation, or diabetes (Kang and Tanna, 2021). A 2014 systematic review estimated the global prevalence of glaucoma to be 64.3 million and projected this would rise to 111.8 million by 2040 due to increasing life expectancy and an aging population (Tham et al., 2014). The global prevalence of primary open-angle glaucoma (POAG) is 3.05%, while primary angle-closure glaucoma (PACG) is 0.50% (Tham et al., 2014).

While high IOP is a major risk factor for glaucoma, it is not the only defining criterion (Quigley, 2011; Kang and Tanna, 2021). High IOP is classified as over 21 mmHg, but some patients exhibit IOP levels in the normal range and are diagnosed with normal-tension glaucoma (NTG; Collaborative Normal-Tension Glaucoma Study Group, 1998; Kang and Tanna, 2021). NTG is more common in Asian populations, with the Tajimi study from Japan reporting that 82% of POAG patients had normal IOP and the Namil study completed in South Korea finding a prevalence of 77% (Iwase et al., 2004; Kim et al., 2011). These findings suggest that factors beyond IOP may contribute to some cases of glaucoma pathology. (Arrigo et al., 2021; Kang and Tanna, 2021).

## 3 Neuropathology

### 3.1 The link between the eye and the brain

The eye is increasingly referred to as the “window” to the brain due to its unique accessibility and direct visibility of CNS tissue without a bone barrier. The optic nerve, a white matter tract of the CNS, shares many characteristics with other white matter tracts; it contains CNS glial cells such as oligodendrocytes, astrocytes and microglia, and is enclosed within the blood-brain barrier and meninges (Lawlor et al., 2018). Structural and pathological similarities suggest the eye could be used as a diagnostic tool for brain disorders. For example, amyloid beta plaques, a pathological sign of Alzheimer’s disease (AD), have been found in post-mortem retinas of AD patients, and fundoscopy in a transgenic mouse model of AD shows retinal plaque accumulation preceding brain formation (Hart et al., 2016; Koronyo-Hamaoui et al., 2011). Furthermore, optical coherence tomography (OCT) revealed RNFL thinning in AD patients, a characteristic also seen in glaucoma (Alasil et al., 2014; Ascaso et al., 2014). Visual impairments are also noted in AD such as reduced contrast sensitivity, lower visual acuity, colour perception, and vision integration (Polo et al., 2017; Salobrar-García et al., 2019).

Glaucoma shares some key characteristics with neurodegenerative diseases such as AD and Parkinson’s disease, including age-related risk, genetic predisposition, gradual onset, and progressive deterioration (Arrigo et al., 2021). It also involves similar mechanisms such as neuroinflammation and oxidative stress (Izzotti et al., 2003; Bosco et al., 2011). For example, glaucoma mouse models revealed increased microglia activation in the retina and optic nerve, causing damage by releasing pro-inflammatory cytokines and reactive oxygen species (Bosco et al., 2011). Oxidative stress is further noted as glaucoma patients have significantly higher oxidative deoxyribonucleic acid damage in the trabecular meshwork (Izzotti et al., 2003).

### 3.2 Neural degeneration

Glaucomatous brain damage may result from transsynaptic neural degeneration, a process observed in other neurodegenerative diseases, where damage spreads from one neuron to synaptically linked distant neurons due to lack of stimulation and trophic support (Yücel and Gupta, 2008). Transsynaptic degeneration can be anterograde, where a postsynaptic neuron degenerates after damage to its upstream neuron, or retrograde, where a presynaptic neuron degenerates due to injury in its downstream target (Lawlor et al., 2018).

Damage to the RGCs in glaucoma can trigger anterograde degeneration along the visual pathway. The RGC axons exit the retina through the optic nerve, travel through the optic tract and mostly terminate in the lateral geniculate nucleus (LGN), with few projections to the superior colliculus and the suprachiasmatic nucleus (SCN) (Yücel and Gupta, 2008). The LGN has six layers; the two ventral magnocellular layers process motion, and the four dorsal parvocellular layers receive red-green colour information (Yücel, 2003). The koniocellular neurons, between these layers, process blue-yellow colour information (Yücel, 2003; Yücel and Gupta, 2008). Approximately 80% of LGN neurons project to the primary visual cortex, while the rest remain in the LGN (Yücel, 2003; Yücel and Gupta, 2008). Damage to the RGCs in glaucoma may propagate throughout the visual pathway, to the LGN and visual cortex (Lawlor et al., 2018). While it is possible that neurodegeneration originates in the brain and precedes retinal thinning, most evidence supports that posterior visual pathway degeneration follows RGC death (Buckingham, 2008; Calkins and Horner, 2012).

### 3.3 Structural changes

Structural changes in the LGN and visual cortex have been observed in non-human models of glaucoma. Primate studies with experimentally induced high IOP glaucoma revealed neuronal loss of both the magnocellular and parvocellular layers, with a more pronounced effect on the magnocellular neurons in some studies (Crawford et al., 2000; Weber et al., 2000; Yücel et al., 2000; Yücel et al., 2001). A linear relationship was noted between RGC loss and shrinkage of magnocellular and parvocellular cells (Yücel et al., 2001). Metabolism of these cell types was also reduced as disease severity increased (Crawford et al., 2000). Additionally, koniocellular neurons exhibited decreased expression of the postsynaptic density protein, calmodulin-dependent kinase type II-alpha, specific to these neurons (Yücel, 2003). Furthermore, in murine models, glaucoma was associated with heightened excitability of the thalamocortical neurons (TC) and altered synaptic function in the dorsal LGN (Smith et al., 2022; Van Hook et al., 2021). These findings demonstrate that glaucoma impacts all three pathways of the LGN; however, since these studies investigated high IOP-induced glaucoma, their relevance to NTG pathology is limited.

The first evidence of structural changes in humans with POAG was from a 2006 post-mortem study that compared the brain and optic nerve in NTG patients to age-matched controls (Gupta et al., 2006). As expected, the glaucomatous eye exhibited significant optic nerve atrophy, but interestingly, the LGN had volume loss, and the magnocellular and parvocellular neurons appeared smaller in glaucoma patients (Gupta et al., 2006). *In vivo* studies using magnetic resonance imaging (MRI) also found LGN atrophy in NTG patients, which strongly correlates with RNFL thinning (Zhang et al., 2012; Schmidt et al., 2018). However, a limitation of the 2018 study was a lack of age-matched controls, introducing possible bias (Schmidt et al., 2018).

Additionally, MRI studies have revealed alterations in gray matter throughout most of the visual system in POAG patients (Frezzotti et al., 2014). For example, advanced-stage POAG patients have significant bilateral cortical thinning of the visual cortex in the anterior calcarine sulci (Boucard et al., 2009; Li et al., 2012; Yu et al., 2013; Bogorodzki et al., 2014; Zhang et al., 2015). This visual cortex thinning correlates with RNFL thinning and visual field deficits, reinforcing the link between RGC degeneration and brain atrophy (Boucard et al., 2009; Yu et al., 2013). Additionally, POAG patients exhibit decreased bilateral gray-matter volume in the occipital cortex, except in the occipital pole corresponding to the central retina, possibly due to preserved central vision in the subjects (Chen et al., 2013). Other occipital lobe regions with reduced gray-matter volume include the lingual gyrus, calcarine gyrus, right cuneus, and right inferior occipital gyrus (Chen et al., 2013). Further investigations using diffuse tensor imaging revealed decreased fractional anisotropy in the inferior fronto-occipital fascicle and inferior longitudinal fascicle, suggesting reduced integrity of these white matter tracts (Frezzotti et al., 2014; Giorgio et al., 2018).

Structural changes in glaucoma extend beyond the visual system, affecting multiple brain regions involved in sensory integration and higher-order processing. Brain atrophy has been identified in the frontoparietal cortex, hippocampus, and cerebellar cortex of glaucoma patients (Frezzotti et al., 2014). Nonvisual white matter tracts with decreased fractional anisotropy include the superior longitudinal fascicle, anterior thalamic radiation, corticospinal tract and middle cerebellar peduncle (Frezzotti et al., 2014). Other regions with notable decreases in bilateral gray-matter volume were the postcentral gyrus, superior frontal gyrus, inferior frontal gyrus, rolandic operculum, left paracentral lobule and right supramarginal gyrus (Chen et al., 2013).

Interestingly some regions in POAG patients exhibit increased gray matter volume compared to individuals who do not have glaucoma. These include the middle temporal gyrus, inferior parietal gyrus, angular gyrus, left superior parietal gyrus, precuneus, and middle occipital gyrus (Chen et al., 2013). Another study identified five structures that were significantly larger for glaucoma patients; right inferior temporal gyrus, right middle occipital gyrus, right occipital lobe white matter, and both inferior occipital gyri (Williams et al., 2013). These regions are involved in high order processing of visual input through the ventral stream (Williams et al., 2013). However, this study only investigated patients with early to moderately advanced glaucoma (Williams et al., 2013). In the moderately advanced group, volume decreases were noted in the right superior frontal gyrus and corpus callosum compared to the control group (Williams et al., 2013). These findings suggest that there may be an initial increase in volume for some structures, but as disease severity increases, brain regions atrophy (Williams et al., 2013).

A possible reason for the volumetric increase of gray matter is cerebral plasticity, where the brain undergoes reorganization to compensate for reduced visual input (Chen et al., 2013). This phenomenon may also be noted in blind individuals where the hippocampus, a region responsible for spatial processing, enlarges, possibly as an adaptive response to vision loss (Fortin et al., 2008). Similarly, studies show that areas within the occipital cortex of blind individuals become activated during the processing of non-visual information, aiding in tactile, auditory and lingual function (Merabet and Pascual-Leone, 2010) Therefore, neuroplasticity may explain the structural alterations in regions within and beyond the visual pathway. Brain regions such as the temporal and parietal lobes may enlarge to compensate for the loss of visual input, while structures traditionally involved in vision enlarge because they are recruited for non-visual tasks (Chen et al., 2013).

### 3.4 Cognitive function and quality of life

The identification of glaucoma-induced neurodegeneration has raised interest in its connection to cognitive function. Studies show glaucoma patients experience deterioration of memory, language, orientation, and higher-order functioning such as judgement and problem-solving (Harrabi et al., 2015; Maurano et al., 2018; Varin et al., 2020; Vidal et al., 2020). For example, glaucoma patients scored lower in verbal working memory and encoding tests than the control group (Varin et al., 2020). Similarly, another study identified lower verbal fluency scores among glaucoma patients (Vidal et al., 2020). Notably, cognitive function scores in individuals with glaucoma were comparable to those with mild AD, and in some cases, those with advanced AD (Maurano et al., 2018). A retrospective cohort study further revealed a 1.21-fold increased dementia risk in POAG patients, though PACG was not associated with an increased risk (Su et al., 2016). Similarly, a systematic review noted that those diagnosed with glaucoma had a significantly increased risk of developing AD (Xu et al., 2019). The correlation between cognitive impairment and glaucoma suggests that glaucomatous damage extends beyond the eye and the visual pathway to impact various structures of the brain. However, limitations such as confounding factors, inconsistent diagnosis criteria and recall or selection bias, particularly in case-control studies, hinder the ability to find a true association between glaucoma and AD risk. (Xu et al., 2019).

There are various negative implications for one’s quality of life from the effects of glaucoma. Firstly, vision loss may lead to a less active lifestyle and decreased cognitive and social stimulation (Varin et al., 2017). Some researchers propose that cognitive decline in glaucoma may stem from vision loss itself, aside from neurodegeneration. According to the “use it or lose it” hypothesis, decreased cognitive engagement leads to atrophy of those processes and skills (Hultsch et al., 1999). A cross-sectional study in Canada reported that individuals with glaucoma participated in 1.8 fewer cognitive activities per month compared to age-matched controls with normal vision (Varin et al., 2017). Therefore, vision loss from glaucoma may contribute to increased risk of cognitive impairment from decreased brain stimulation and engagement in activities (Varin et al., 2017).

Glaucoma may also negatively impact quality of life by altering the circadian rhythm. Intrinsically photosensitive retinal ganglion cells (ipRGCs) transmit photic signals to the SCN via the retinohypothalmic tract (Gubin et al., 2024). The SCN acts as the central pacemaker for circadian rhythm regulation and coordinates several physiological and behavioral processes (Gubin et al., 2024). IpRGC dysfunction has been observed in moderate and severe glaucoma, with greater disease severity correlating with worsening function (Feigl et al., 2011; Kankipati et al., 2011). Damage to the ipRGCs reduces signal transmission to the SCN, impairing the brain’s ability to process light cues (Gubin et al., 2024). One study linked RGC global loss volume to altered circadian temperature rhythm, but did not investigate ipRGCs specifically (Gubin et al., 2019). Additionally, the higher resistance to injury of some classes of ipRGCs over other RGCs adds uncertainty to the impact of ipRGC loss on circadian disturbance (Gubin et al., 2019).

Studies have also evaluated sleep quality in glaucoma patients. Those with glaucoma experienced lower average total sleep time, reduced sleep efficiency, higher arousal duration, and increased periodic limb movements during sleep (Gracitelli et al., 2015; Gubin et al., 2019). Additionally, sleep disturbances were higher in POAG and PACG patients compared to age-matched controls (Wang et al., 2013) and glaucoma patients were also reported to exhibit higher daytime sleepiness (Gracitelli et al., 2016). Beyond sleep disruptions, impaired circadian rhythms in glaucoma can negatively affect mood regulation, antioxidant defense, immune function and metabolism (Lazzerini Ospri et al., 2017; Gubin et al., 2024). Interestingly, oral melatonin supplementation has shown potential in alleviating these disruptions, improving RGC function, sleep quality, and mood for patients with advanced glaucoma (Gubin et al., 2021). Given the substantial impact of glaucoma on circadian regulation, treatment strategies should account for these effects to enhance patients’ quality of life.

## 4 Discussion

Glaucoma exhibits characteristics of a neurodegenerative disorder that impacts brain structure, cognitive function, and quality of life. Glaucoma-induced RGC death triggers neurodegeneration that extends beyond the eye, involving the LGN, visual cortex, and other brain regions (Gupta et al., 2006; Frezzotti et al., 2014). It can also disrupt circadian rhythm, leading to decreased sleep quality, mood alterations, and metabolic dysfunction (Gubin et al., 2024). These widespread effects highlight the need for research on glaucoma’s impact through the entire visual pathway and the development of comprehensive treatment strategies. Promising therapies include anti-inflammatory, antioxidant and neuroprotective agents. For example, crocetin has been shown to restore optic nerve and retina structure, reduce inflammatory factors, and increase brain-derived neurotrophic factors and Nissl bodies in the primary visual cortex in glaucoma rats (Li et al., 2023). Similarly, ginkgo biloba extract in combination with docosahexanoic acid restored hippocampal tissue damage and improved cognitive memory and spatial learning in AD-induced mice (Abdelmeguid et al., 2021), and when administered alone, ginkgo biloba improved visual field damage in NTG patients (Quaranta et al., 2003).

Furthermore, the impact of cognitive decline should be considered in glaucoma diagnosis and treatment. Visual field tests used for glaucoma diagnosis are less reliable with cognitive decline, potentially affecting accurate disease management (Diniz-Filho et al., 2017). In terms of treatment, cognitive deficits may reduce patient adherence to glaucoma medications and must be accounted for when providing treatment options (Maurano et al., 2018).

A major limitation of existing literature is the lack of longitudinal studies. Most findings are cross-sectional and can only establish a correlation between glaucoma and structural brain changes. As a result, it remains unclear whether RGC degeneration leads to brain atrophy or if neurodegenerative processes originating in the brain contribute to glaucoma progression. Longitudinal studies are also needed to determine if glaucoma patients display lower cognitive function due to performing fewer cognitive activities. Furthermore, some studies failed to include age-matched controls, raising the possibility that observed structural brain changes are due to normal aging or other age-related neurodegenerative conditions rather than glaucoma pathology. Another limitation is the reliance on subjective measures in certain studies, such as those evaluating sleep quality using a questionnaire. Self-reported sleep disturbances may be influenced by various factors, making it difficult to isolate the direct effects of glaucoma on circadian regulation.

## References

[B1] Abdelmeguid N. E. Khalil M. I. M. Elhabet R. Sultan A. S. Salam S. A. (2021). Combination of docosahexaenoic acid and ginko biloba extract improves cognitive function and hippocampal tissue damages in a mouse model of Alzheimer’s disease. J. Chem. Neuroanat. 116, 101995. 10.1016/j.jchemneu.2021.101995 34182090

[B2] Alasil T. Wang K. Yu F. Field M. G. Lee H. Baniasadi N. (2014). Correlation of retinal nerve fiber layer thickness and visual fields in glaucoma: a broken stick model. Am. J. Ophthalmol. 157 (5), 953–959. 10.1016/j.ajo.2014.01.014 24487047 PMC4423422

[B3] Arrigo A. Aragona E. Saladino A. Arrigo D. Fantaguzzi F. Battaglia Parodi M. (2021). Cognitive dysfunctions in glaucoma: an overview of morpho-functional mechanisms and the impact on higher-order visual function. Front. Aging Neurosci. 13, 747050. 10.3389/fnagi.2021.747050 34690746 PMC8526892

[B4] Ascaso F. J. Cruz N. Modrego P. J. Lopez-Anton R. Santabárbara J. Pascual L. F. (2014). Retinal alterations in mild cognitive impairment and Alzheimer’s disease: an optical coherence tomography study. J. Neurology 261 (8), 1522–1530. 10.1007/s00415-014-7374-z 24846203

[B5] Bogorodzki P. Piątkowska-Janko E. Szaflik J. Szaflik J. P. Gacek M. Grieb P. (2014). Mapping cortical thickness of the patients with unilateral end-stage open angle glaucoma on planar cerebral cortex maps. PLoS ONE 9 (4), e93682. 10.1371/journal.pone.0093682 24709970 PMC3977872

[B6] Bosco A. Steele M. R. Vetter M. L. (2011). Early microglia activation in a mouse model of chronic glaucoma. J. Comp. Neurology 519 (4), 599–620. 10.1002/cne.22516 PMC4169989 21246546

[B7] Boucard C. C. Hernowo A. T. Maguire R. P. Jansonius N. M. Roerdink J. B. T. M. Hooymans J. M. M. (2009). Changes in cortical grey matter density associated with long-standing retinal visual field defects. Brain a J. neurology 132 (Pt 7), 1898–1906. 10.1093/brain/awp119 PMC2702836 19467992

[B8] Buckingham B. P. Inman D. M. Lambert W. Oglesby E. Calkins D. J. Steele M. R. (2008). Progressive ganglion cell degeneration precedes neuronal loss in a mouse model of glaucoma. J. Neurosci. 28 (11), 2735–2744. 10.1523/JNEUROSCI.4443-07.2008 18337403 PMC6670674

[B9] Calkins D. J. Horner P. J. (2012). The cell and molecular biology of glaucoma: axonopathy and the brain. Investigative Opthalmology and Vis. Sci. 53 (5), 2482–2484. 10.1167/iovs.12-9483i PMC3998097 22562846

[B10] Chen W. W. Wang N. Cai S. Fang Z. Yu M. Wu Q. (2013). Structural brain abnormalities in patients with primary open-angle glaucoma: a study with 3T MR imaging. Investigative Ophthalmol. Vis. Sci. 54 (1), 545–554. 10.1167/iovs.12-9893 23258150

[B11] Collaborative Normal-Tension Glaucoma Study Group (1998). The effectiveness of intraocular pressure reduction in the treatment of normal-tension glaucoma. Collaborative normal-tension glaucoma study group. Am. J. Ophthalmol. 126 (4), 498–505. 10.1016/S0002-9394(98)00272-4 9780094

[B12] Crawford M. L. J. Harwerth R. S. Smith E. L. 3rd Shen F. Carter-Dawson L. (2000). Glaucoma in primates: cytochrome oxidase reactivity in parvo-and magnocellular pathways. Invest Ophthalmol. Vis. Sci., 1791–1802. 10845600

[B13] Diniz-Filho A. Delano-Wood L. Daga F. B. Cronemberger S. Medeiros F. A. (2017). Association between neurocognitive decline and visual field variability in glaucoma. JAMA Ophthalmol. 135 (7), 734–739. 10.1001/jamaophthalmol.2017.1279 28520873 PMC5710202

[B14] Feigl B. Mattes D. Thomas R. Zele A. J. (2011). Intrinsically photosensitive (melanopsin) retinal ganglion cell function in glaucoma. Investigative Ophthalmol. Vis. Sci. 52 (7), 4362–4367. 10.1167/iovs.10-7069 21498620

[B15] Fortin M. Voss P. Lord C. Lassonde M. Pruessner J. Saint-Amour D. (2008). Wayfinding in the blind: larger hippocampal volume and supranormal spatial navigation. Brain 131 (11), 2995–3005. 10.1093/brain/awn250 18854327

[B16] Frezzotti P. Giorgio A. Motolese I. De Leucio A. Iester M. Motolese E. (2014). Structural and functional brain changes beyond visual system in patients with advanced glaucoma. PLoS ONE 9 (8), e105931. 10.1371/journal.pone.0105931 25162716 PMC4146554

[B17] Giorgio A. Zhang J. Costantino F. De Stefano N. Frezzotti P. (2018). Diffuse brain damage in normal tension glaucoma. Hum. Brain Mapp. 39 (1), 532–541. 10.1002/hbm.23862 29064608 PMC6866372

[B18] Gracitelli C. P. B. Duque-Chica G. L. Moura A. L. d. A. Roizenblatt M. Nagy B. V. de Melo G. R. (2016). Relationship between daytime sleepiness and intrinsically photosensitive retinal ganglion cells in glaucomatous disease. J. Ophthalmol. 2016, 5317371–5317379. 10.1155/2016/5317371 26955483 PMC4756205

[B19] Gracitelli C. P. B. Duque-Chica G. L. Roizenblatt M. Moura A. L. d. A. Nagy B. V. Ragot de Melo G. (2015). Intrinsically photosensitive retinal ganglion cell activity is associated with decreased sleep quality in patients with glaucoma. Ophthalmology 122 (6), 1139–1148. 10.1016/j.ophtha.2015.02.030 25858174

[B20] Gubin D. Malishevskaya T. Weinert D. Zakharova E. Astakhov S. Cornelissen G. (2024). “Circadian disruption in glaucoma: causes, consequences, and countermeasures. Front. Biosci. - Landmark, 29 (12), 410. 10.31083/j.fbl2912410 39735989

[B21] Gubin D. Neroev V. Malishevskaya T. Cornelissen G. Astakhov S. Y. Kolomeichuk S. (2021). Melatonin mitigates disrupted circadian rhythms, lowers intraocular pressure, and improves retinal ganglion cells function in glaucoma. J. Pineal Res. 70 (4), e12730. 10.1111/jpi.12730 33730443

[B22] Gubin D. G. Malishevskaya Т. N. Astakhov Y. S. Astakhov S. Y. Cornelissen G. Kuznetsov V. A. (2019). Progressive retinal ganglion cell loss in primary open-angle glaucoma is associated with temperature circadian rhythm phase delay and compromised sleep. Chronobiology Int. 36 (4), 564–577. 10.1080/07420528.2019.1566741 30663431

[B23] Gupta N. Noël de Tilly L. Bidaisee L. Yücel Y. H. (2006). Human glaucoma and neural degeneration in intracranial optic nerve, lateral geniculate nucleus, and visual cortex. Br. J. Ophthalmol. 90 (6), 674–678. 10.1136/bjo.2005.086769 16464969 PMC1860237

[B24] Harrabi H. Kergoat M. J. Rousseau J. Boisjoly H. Schmaltz H. Moghadaszadeh S. (2015). Age-related eye disease and cognitive function. Investigative Ophthalmol. Vis. Sci. 56 (2), 1217–1221. 10.1167/iovs.14-15370 25650424

[B25] Hart N. J. Koronyo Y. Black K. L. Koronyo-Hamaoui M. (2016). Ocular indicators of Alzheimer’s: exploring disease in the retina. Acta Neuropathol. 132, 767–787. 10.1007/s00401-016-1613-6 27645291 PMC5106496

[B26] Hultsch D. F. Hertzog C. Small B. J. Dixon R. A. (1999). Use it or lose it: engaged lifestyle as a buffer of cognitive decline in aging? Psychol. Aging 14 (2), 245–263. 10.1037/0882-7974.14.2.245 10403712

[B27] Iwase A. Suzuki Y. Araie M. Yamamoto T. Abe H. Shirato S. (2004). The prevalence of primary open-angle glaucoma in Japanese: the tajimi study. Ophthalmology 111 (9), 1641–1648. 10.1016/j.ophtha.2004.03.029 15350316

[B28] Izzotti A. Saccà S. C. Cartiglia C. De Flora S. (2003). Oxidative deoxyribonucleic acid damage in the eyes of glaucoma patients. Am. J. Med. 114 (8), 638–646. 10.1016/S0002-9343(03)00114-1 12798451

[B29] Kang J. M. Tanna A. P. (2021). Glaucoma. Med. Clin. N. Am. 105 (3), 493–510. 10.1016/j.mcna.2021.01.004 33926643

[B30] Kankipati L. Girkin C. A. Gamlin P. D. (2011). The post-illumination pupil response is reduced in glaucoma patients. Investigative Ophthalmol. and Vis. Sci. 52 (5), 2287–2292. 10.1167/iovs.10-6023 PMC3080733 21212172

[B31] Kass M. A. Heuer D. K. Higginbotham E. J. Johnson C. A. Keltner J. L. Miller J. P. (2002). The ocular hypertension treatment study: a randomized trial determines that topical ocular hypotensive medication delays or prevents the onset of primary open-angle glaucoma. Arch. Ophthalmol. 120 (6), 701–713. 10.1001/archopht.120.6.701 12049574

[B32] Kim C. Seong G. J. Lee N. h. Song K. c. Namil Study Group, Korean Glaucoma Society (2011). Prevalence of primary open-angle glaucoma in central South Korea: the namil study. Ophthalmology 118 (6), 1024–1030. 10.1016/j.ophtha.2010.10.016 21269703

[B33] Kim K. E. Park K.-H. (2016). Update on the prevalence, etiology, diagnosis, and monitoring of normal-tension glaucoma. Asia-Pacific J. Ophthalmol. 5 (1), 23–31. 10.1097/APO.0000000000000177 26886116

[B34] Koronyo-Hamaoui M. Koronyo Y. Ljubimov A. V. Miller C. A. Ko M. K. Black K. L. (2011). Identification of amyloid plaques in retinas from Alzheimer’s patients and noninvasive *in vivo* optical imaging of retinal plaques in a mouse model. NeuroImage 54, S204–S217. 10.1016/j.neuroimage.2010.06.020 20550967 PMC2991559

[B35] Lawlor M. Danesh-Meyer H. Levin L. A. Davagnanam I. De Vita E. Plant G. T. (2018). Glaucoma and the brain: trans-Synaptic degeneration, structural change, and implications for neuroprotection. Surv. Ophthalmol. 63 (3), 296–306. 10.1016/j.survophthal.2017.09.010 28986311

[B36] Lazzerini Ospri L. Prusky G. Hattar S. (2017). Mood, the circadian system, and melanopsin retinal ganglion cells. Annu. Rev. Neurosci. 40 (1), 539–556. 10.1146/annurev-neuro-072116-031324 28525301 PMC5654534

[B37] Li C. Cai P. Shi L. Lin Y. Zhang J. Liu S. (2012). Voxel-based morphometry of the visual-related cortex in primary open angle glaucoma. Curr. Eye Res. 37 (9), 794–802. 10.3109/02713683.2012.683506 22631870

[B38] Li Q. Feng P. Lin S. Xu Z. Zhao J. Chen Z. (2023). Crocetin confers neuroprotection and is anti-inflammatory in rats with induced glaucoma. Mol. Biol. Rep. 50 (2), 1321–1331. 10.1007/s11033-022-08102-9 36456771

[B39] Maurano S. T. P. da Silva D. J. Ávila M. P. Magacho L. (2018). Cognitive evaluation of patients with glaucoma and its comparison with individuals with Alzheimer’s disease. Int. Ophthalmol. 38 (5), 1839–1844. 10.1007/s10792-017-0658-4 28744790

[B40] Merabet L. B. Pascual-Leone A. (2010). Neural reorganization following sensory loss: the opportunity of change. Nat. Rev. Neurosci. 11 (1), 44–52. 10.1038/nrn2758 19935836 PMC3898172

[B41] Polo V. Rodrigo M. J. Garcia-Martin E. Otin S. Larrosa J. M. Fuertes M. I. (2017). Visual dysfunction and its correlation with retinal changes in patients with Alzheimer’s disease. Eye 31 (7), 1034–1041. 10.1038/eye.2017.23 28282060 PMC5519267

[B42] Quaranta L. Bettelli S. Uva M. G. Semeraro F. Turano R. Gandolfo E. (2003). Effect of Ginkgo biloba extract on preexisting visual field damage in normal tension glaucoma. Ophthalmology 110 (2), 359–362. 10.1016/S0161-6420(02)01745-1 12578781

[B43] Quigley H. A. (2011). Glaucoma. Lancet 377 (9774), 1367–1377. 10.1016/S0140-6736(10)61423-7 21453963

[B44] Salobrar-García E. de Hoz R. Ramírez A. I. López-Cuenca I. Rojas P. Vazirani R. (2019). Changes in visual function and retinal structure in the progression of Alzheimer’s disease. PLOS ONE 14 (8), e0220535. 10.1371/journal.pone.0220535 31415594 PMC6695171

[B45] Schmidt M. A. Knott M. Heidemann R. Michelson G. Kober T. Dörfler A. (2018). Investigation of lateral geniculate nucleus volume and diffusion tensor imaging in patients with normal tension glaucoma using 7 tesla magnetic resonance imaging. PLOS ONE 13 (6), e0198830. 10.1371/journal.pone.0198830 29879191 PMC5991727

[B46] Smith J. C. Zhang K. Y. Sladek A. Thompson J. Bierlein E. R. Bhandari A. (2022). Loss of retinogeniculate synaptic function in the DBA/2J mouse model of glaucoma. eNeuro 9 (6), ENEURO.0421–ENEURO.0422. 10.1523/ENEURO.0421-22.2022 PMC9794376 36526366

[B47] Su C.-W. Lin C. C. Kao C. H. Chen H. Y. (2016). Association between glaucoma and the risk of dementia. Medicine 95 (7), e2833. 10.1097/MD.0000000000002833 26886642 PMC4998642

[B48] Tham Y.-C. Li X. Wong T. Y. Quigley H. A. Aung T. Cheng C. Y. (2014). Global prevalence of glaucoma and projections of glaucoma burden through 2040: a systematic review and meta-analysis. Ophthalmology 121 (11), 2081–2090. 10.1016/j.ophtha.2014.05.013 24974815

[B49] Van Hook M. J. Monaco C. Bierlein E. R. Smith J. C. (2021). Neuronal and synaptic plasticity in the visual thalamus in mouse models of glaucoma. Front. Cell. Neurosci. 14, 626056. 10.3389/fncel.2020.626056 33584206 PMC7873902

[B50] Varin M. Kergoat M. J. Belleville S. Li G. Rousseau J. Roy-Gagnon M. H. (2017). Age-related eye disease and participation in cognitive activities. Sci. Rep. 7 (1), 17980. 10.1038/s41598-017-18419-2 29269882 PMC5740122

[B51] Varin M. Kergoat M. J. Belleville S. Li G. Rousseau J. Roy-Gagnon M. H. (2020). Age-related eye disease and cognitive function: the search for mediators. Ophthalmology 127 (5), 660–666. 10.1016/j.ophtha.2019.10.004 31727427

[B52] Vidal K. S. Suemoto C. K. Moreno A. B. Duncan B. Schmidt M. I. Maestri M. (2020). Association between cognitive performance and self-reported glaucoma in middle-aged and older adults: a cross-sectional analysis of ELSA-brasil. Braz. J. Med. Biol. Res. 53 (12), e10347. 10.1590/1414-431x202010347 33146284 PMC7643934

[B53] Wang H. Zhang Y. Ding J. Wang N. (2013). Changes in the circadian rhythm in patients with primary glaucoma. PLoS ONE 8 (4), e62841. 10.1371/journal.pone.0062841 23658653 PMC3639222

[B54] Weber A. J. Chen H. Hubbard W. C. Kaufman P. L. (2000). Experimental glaucoma and cell size, density, and number in the primate lateral geniculate nucleus. Investigative Ophthalmol. and Vis. Sci. 41 (6), 1370–1379. 10798652

[B55] Williams A. L. Lackey J. Wizov S. S. Chia T. M. T. Gatla S. Moster M. L. (2013). Evidence for widespread structural brain changes in glaucoma: a preliminary voxel-based MRI study. Investigative Opthalmology and Vis. Sci. 54 (8), 5880–5887. 10.1167/iovs.13-11776 23838767

[B56] Xu X. H. Zou J.-Y. Geng W. (2019). Association between glaucoma and the risk of Alzheimer’s disease: a systematic review of observational studies. Acta Ophthalmol., 665–671. 10.1111/aos.14114 31012234

[B57] Yu L. Xie B. Yin X. Liang M. Evans A. C. Wang J. (2013). Reduced cortical thickness in primary open-angle glaucoma and its relationship to the retinal nerve fiber layer thickness. PLoS ONE 8 (9), e73208. 10.1371/journal.pone.0073208 24019910 PMC3760921

[B58] Yücel Y. Gupta N. (2008). Glaucoma of the brain: a disease model for the study of transsynaptic neural degeneration. Prog. Brain Res. 173, 465–478. 10.1016/S0079-6123(08)01132-1 18929128

[B59] Yücel Y. Zhang Q. Weinreb R. N. Kaufman P. L. Gupta N. (2003). Effects of retinal ganglion cell loss on magno-parvo-koniocellular pathways in the lateral geniculate nucleus and visual cortex in glaucoma. Prog. Retin. Eye Res. 22 (4), 465–481. 10.1016/S1350-9462(03)00026-0 12742392

[B60] Yücel Y. H. Zhang Q. Weinreb R. N. Kaufman P. L. Gupta N. (2001). Atrophy of relay neurons in magno-and parvocellular layers in the lateral geniculate nucleus in experimental glaucoma. Invest. Ophthalmol. Vis. Sci. 3216–3222. 11726625

[B61] Yücel Y. H. Zhang Q. Gupta N. Kaufman P. L. Weinreb R. N. (2000). Loss of neurons in magnocellular and parvocellular layers of the lateral geniculate nucleus in glaucoma. Archives Ophthalmol. 118 (3), 378–384. 10.1001/archopht.118.3.378 10721961

[B62] Zhang S. Wang B. Xie Y. Zhu S. Thomas R. Qing G. (2015). Retinotopic changes in the gray matter volume and cerebral blood flow in the primary visual cortex of patients with primary open-angle glaucoma. Investigative Ophthalmol. Vis. Sci. 56 (10), 6171–6178. 10.1167/iovs.15-17286 26406275

[B63] Zhang Y. Q. Li J. Xu L. Zhang L. Wang Z. C. Yang H. (2012). Anterior visual pathway assessment by magnetic resonance imaging in normal‐pressure glaucoma. Acta Ophthalmol. 90 (4), e295–e302. 10.1111/j.1755-3768.2011.02346.x 22489916

